# Clinical features and prognosis of prosthetic valve endocarditis due to *Staphylococcus aureus*

**DOI:** 10.1007/s10096-024-04848-1

**Published:** 2024-08-07

**Authors:** Itziar Diego-Yagüe, Antonio Ramos-Martínez, Patricia Muñoz, Manuel Martínez-Sellés, Marina Machado, Arístides de Alarcón, José M. Miró, Raquel Rodríguez-Gacía, José Francisco Gutierrez-Díez, Carmen Hidalgo-Tenorio, Belén Loeches-Yagüe, Juan Carlos López-Azor

**Affiliations:** 1 Unidad de Enfermedades Infecciosas, Hospital Universitario Puerta de Hierro, IDIPHISA, Majadahonda, Madrid, Spain; 2 Universidad Autónoma de Madrid, Madrid, Spain; 3 Unidad de Enfermedades Infecciosas, Servicio de Medicina Interna, Universitario Puerta de Hierro, Majadahonda, Madrid, Madrid, Spain; 4 Servicio de Microbiología Clínica y Enfermedades Infecciosas, Madrid, Spain; 5 Instituto de Investigación Sanitaria Gregorio Marañón. CIBER Enfermedades Respiratorias-CIBERES, Facultad de Medicina, Universidad Complutense de Madrid, Madrid, Spain; 6 Servicio de Cardiología, Hospital General Universitario Gregorio Marañón, Madrid, Spain; 7 CIBERCV, Universidad Europea, Universidad Complutense, Madrid, Spain; 8 Instituto de Investigación Sanitaria Gregorio Marañón, Madrid, Spain; 9 Unidad Clínica de Enfermedades Infecciosas, Microbiología y Parasitología (UCEIMP), Instituto de Biomedicina de Sevilla (IBiS), Hospital Universitario Virgen del Rocío, Sevilla, Spain; 10 Servicio de Enfermedades Infecciosas. Hospital Clínic-IDIBAPS, Universidad de Barcelona, Barcelona, Spain; 11 Servicio de Medicina Intensiva, Hospital Universitario Central de Asturias, Universidad de Oviedo, Oviedo, Spain; 12 Servicio de Cirugía Cardiovascular, Hospital Universitario Marqués de Valdecilla Santander, Cantabria, Spain; 13 Servicio de Medicina Interna, Hospital Universitario Virgen de las Nieves, IBS-Granada, Granada, Spain; 14 Unidad de Enfermedades Infecciosas, Hospital Universitario La Paz, Madrid, Spain; 15 CIBERINFEC, Instituto de Salud Carlos III, Madrid, Spain; 16 Servicio de Cardiología, Hospital Universitario Puerta de Hierro, Majadahonda, Madrid, Spain

**Keywords:** Heart valve prosthesis, Endocarditis, Staphylococcus aureus, Methicillin resistance, Cardiac surgical procedures, Mortality

## Abstract

**Purpose:**

*Staphylococcus aureus* prosthetic valve endocarditis (SAPVE) is a serious infection with high mortality. The main objective of this study was to identify factors associated with in-hospital mortality.

**Methods:**

From January 2008 to December 2021, consecutive patients from a Spanish cohort of infective endocarditis with a definitive diagnosis of SAPVE were analyzed.

**Results:**

During the study period, 219 cases of definitive SAPVE were diagnosed, which accounted for 16.7% of a total of 1309 cases of definitive prosthetic valve endocarditis (PVE). Patients presented advanced age and marked comorbidity. There was a higher incidence of persistent bacteremia, septic shock, stroke, and acute kidney injury than in cases of PVE caused by other microorganisms. Methicillin resistance was not associated with differences in clinical presentation, echocardiographic findings, or mortality. Only 50.6% of the patients with surgical indications (88 patients) underwent surgery. Overall, in-hospital mortality was 47.9%. The variables associated with in-hospital mortality were age (OR:1.03, 95% CI: 1.00-1.05; *p* = 0.016), heart failure (OR:2.86, 95% CI: 1.53–5.32; *p* = 0.001), acute kidney injury (OR:2.42, 95%CI:1.28–4.58; *p* = 0.006), stroke (OR:3.53, 95%CI:1.79–6.96; *p* < 0.001) and surgery indicated but not performed (OR:2.01, 95%CI:1.06–3.8; *p* = 0.030). On the other hand, the performance of surgery per se in patients with SAPVE, regardless of whether there was a surgical indication according to the guidelines, was not associated with a reduction in in-hospital mortality.

**Conclusions:**

SAPVE is characterized by high mortality, which is more marked in patients who present a surgical indication but do not undergo surgery.

**Supplementary Information:**

The online version contains supplementary material available at 10.1007/s10096-024-04848-1.

## Introduction

Prosthetic valve endocarditis (PVE) accounts for 20–30% of cases of infective endocarditis (IE) and is associated with high mortality [1]. *Staphylococcus aureus* is the most common pathogen in PVE diagnosed during the first two months after valve prosthesis placement [2]. This temporal proximity to the hospital stay means that a high percentage of cases of SAPVE are caused by methicillin-resistant *S. aureus* (MRSA), whose available antibiotic treatment may be less effective than that used when the pathogen is methicillin-sensitive *S. aureus* (MSSA) [3–5]. Unfortunately, studies that have compared the characteristics of EVP caused by MRSA with those caused by MSSA have been limited and with a reduced number of patients [3, 6–8].

The greater virulence of *S. aureus* compared to other pathogens has been related to the marked severity of PVE due this bacterial species [9]. This severity is clinically manifested by a higher frequency of septic shock, persistent bacteremia, stroke, and perivalvular abscess, among other complications [2, 7, 9–11].

Despite the severity of this condition, several studies have shown that the percentage of patients who undergo surgery is relatively low. Clinical instability or increased surgical complexity, a hallmark of these patients, may be related to the fact that these patients are frequently dismissed for surgical treatment [3, 9].

The objectives of the study were to compare the cases of PVE due to *S. aureus* (SAPVE) with those caused by other pathogens, to determine the clinical characteristics of PVE due to MRSA and to identify the factors associated with hospital mortality. Finally, the aim was to analyze whether surgery performed on all patients with SAPVE, regardless of whether or not there was a surgical indication according to the clinical guidelines, was associated with lower in-hospital mortality.

## Patients and methods

From 1st January 2008 to 31th December 2021, consecutive patients with a definite diagnosis IE, according to Duke’s modified criteria, were prospectively included. These patients received treatment in a group of Spanish hospitals, collectively serving approximately 30% of the nation’s population. At each center, a multidisciplinary team completes a standardized form with the IE episode and a follow-up form after one year of the episode. The register included sections for demographic, clinical, microbiological, echocardiographic, management and prognostic information. The cohort registration received approval of regional and local ethics committees. Specifically, the Ethics and Clinical Research Board of one of participant hospitals approved the study protocol and publication of data (Gregorio Marañón Hospital in Madrid, number 18/07). Written informed consent was obtained in cases where the patient could be adequately informed. In the case of patients unable to give consent, the ethical committees waived investigators from the obligation to obtain consent to avoid bias in patient inclusion. Data and samples were collected from 1st January 2008 to 31th December 2021. The clinical data of patients included in the medical records were accessed for research purposes. Access to medical records containing information that could identify individual participants during data collection was conducted in a manner that protected patient privacy at every point in time. Subsequently, the study data were analyzed during the years 2022 and 2023. The authors did not have access to information that could identify individual participants during or after data collection. The data on which this study is based are available upon reasonable request through the technical office of the research network [(Spanish collaboration on endocarditis (GAMES)] which can be contacted via this e-mail: games08@gmail.com.

### Definitions

#### General variables

General definitions correspond to those published in other studies on endocarditis [12, 13]. Healthcare-associated infections were defined as previously published [14]. Patients were categorized into either early or late PVE, depending on whether the diagnosis was made before or after the first year following prosthetic valve implantation, respectively [15, 16]. Persistent bacteremia was defined as persistence of positive blood cultures after 7 days of appropriate antibiotic treatment initiation. Systemic embolization included embolism to any major arterial vessel, excluding stroke, which was defined by acute neurological deficit of vascular origin lasting > 24 h. Episodes with neurological symptoms lasting less than 24 h, but showing imaging scans suggestive of infarction, were classified as stroke [17].

#### Exposures of interest

Surgical indications followed the latest current European guidelines available at the time of diagnosis [18–20]. Particular focus was directed to identifying patients with surgical indications and, within this group, those who were not operated on.

#### Outcomes of interest

In-hospital mortality and 1-year mortality were defined as death from any cause during hospital admission or within the 365 days following admission in which PVE was treated, respectively. Recurrent IE was defined as a new episode of IE caused by the same or another microorganism during the first year of follow-up.

### Patients

The study analyzed demographic, clinical, echocardiographic, and treatment data of the included patients, as well as morbidity and mortality both at admission and during the first year of follow-up. Patients with atrial or ventricular septal defect closure or cardiovascular implantable electronic devices infection were included only if they had a concomitantly infected prosthetic valve.

### Statistical analysis

Categoric variables are expressed as absolute numbers and percentages. Quantitative variables are expressed as median and interquartile range (IQR). Categorical variables were compared using *χ*^2^ test or Fisher test when necessary. Quantitative variables were compared using Mann-Whitney’s U. In the comparison of risk factors for mortality, those variables with *p* < 0.10 in univariant analysis and that were considered clinically significant, were included in a multivariate logistic regression model, with a maximum of one variable for every 10 events (deaths). The goodness of fit of the final multivariate mode was assessed again by the Hosmer-Lemeshow test. Adjusted odds ratios and its 95% confident interval are provided. Bilateral p-value below 0.05 was considered statistically significant. To better assess the correlation between surgery and in-hospital mortality in PVE due to *S. aureus*, we performed a propensity-score-based analysis. We developed a PS controlled for chronic liver disease, age-adjusted Charlson index, valve vegetation and perivalvular abscess. Calibration of the model was assessed by Hosmer-Lemeshow test. Then, we performed a 1:1 exact matching with no replacement. To assess the balancing between groups we compared absolute differences in baseline and clinical characteristics and compared them using the same univariate method described above. All statistical analyses were performed with SPSS version 25 software (SPSS INC., Chicago, Illinois, USA).

## Results

During the study period, 219 cases of definite SAPVE were diagnosed, which accounted for 16.7% of a total of 1309 cases of definite PVE. Patients were characterized by advanced age [median 69 years, interquartile range (IQR) 61–76 years], being predominantly male (67%), having marked comorbidity with a median Charlson index of 5 (IQR: 5–7), a severe and complicated clinical course, and a high percentage (39.3%) of patients who did not undergo surgery despite having a surgical indication (Table 1). In-hospital mortality was 47.9%. One hundred thirty-five patients (61.6%) had aortic valve infection and 115 patients (52.5%) had mitral valve infection. Thirty-six patients (16.4%) had simultaneous involvement of the aortic and mitral valves.

**Table 1:** Comparison of the characteristics of patients with PVE due to *S. aureus* with those produced by other microorganism

	*S. aureus* (*n* = 219)	Other etiology (*n* = 1171)	*p*	
Age, years (IQR)	69 (61–76)	71 (63–78)	0.019	
Male gender	147 (67.1)	778 (66.4)	0.844	
Community-acquired	118 (53.9)	651 (55.6)	0.640	
Hospital-acquired	89 (40.6)	443 (37.8)	0.433	
Non-nosocomial healthcare related	12 (5.5)	77 (6.6)	0.543	
Early PVE (first year)	449 (38.3)	59 (26.9)	0.001	
Site of infection				
Aortic	135 (61.6)	861 (73.6)	< 0.001	
Transcatheter aortic valve implantation	7 (3.2)	25 (2.1)	0.336	
Mitral	115 (52.5)	410 (35.0)	< 0.001	
Tricuspid	2 (0.9)	14 (1.2)	0.719	
Pulmonary	6 (2.7)	27 (2.3)	0.699	
CIED ^a^	6 (2.7)	18 (1.5)	0.210	
Comorbidity				
Chronic respiratory disease	54 (24.6)	211 (18.0)	0.022	
Coronary disease	79 (36.0)	407 (34.7)	0.708	
Cardiac insufficiency	103 (47.0)	525 (44.8)	0.548	
Diabetes mellitus	66 (30.1)	351 (29.9)	0.962	
Peripheral vascular disease	25 (11.4)	109 (9.3)	0.332	
Cerebrovascular disease	29 (13.2)	212 (18.1)	0.081	
Neoplasia	40 (18.2)	187 (15.9)	0.399	
Chronic renal failure	70 (32.0)	303 (25.9)	0.062	
Chronic liver disease	20 (9.1)	77 (6.6)	0.173	
Congenital heart disease	14 (6.4)	77 (6.6)	0.920	
Age-adjusted Charlson Comorbidity Index (IQR)	5 (3–7)	5 (3–7)	0.516	
Vegetation	158 (72.1)	797 (68.1)	0.231	
Intracardiac complications	73 (33.3)	515 (44.0)	0.011	
Valve perforation or rupture	5 (2.2)	49 (4.1)	0.181	
Pseudoaneurysm	11 (5.0)	139 (11.8)	0.003	
Perivalvular abscess	62 (28.3)	411 (35.1)	0.135	
Intracardiac fistula	9 (4.1)	56 (4.7)	0.665	
Clinical course				
Heart failure	100 (45.6)	457 (39.0)	0.066	
Persistent bacteremia	36 (16.4)	120 (10.2)	0.008	
Stroke	68 (31.0)	260 (22.2)	0.005	
Embolism ^b^	43 (19.6)	248 (21.1)	0.606	
Acute renal injury	131 (59.8)	453 (38.6)	< 0.001	
Septic shock	77 (35.1)	112 (9.5)	< 0.001	
Surgical indication	174 (79.4)	858 (73.3)	0.055	
Surgery performed ^c^	88 (40.2)	574 (49.0)	0.016	
Surgery indicated, not performed	86 (39.3)	290 (24.8)	< 0.001	
Recurrence	6 (2.7)	52 (4.4)	0.248	
In-hospital mortality	105 (47.9)	347 (29.6)	< 0.001	
First year mortality	116 (52.9)	407 (34.7)	< 0.001	

S. aureus: *Staphylococcus aureus*. CIED: Cardiac implantable electronic device. ^a^ Patients with PVE who also harbored CIED. ^b^ Excluding cases with stroke. ^c^ Six patients (0.51%) underwent surgery during the admission in which endocarditis was treated without a specific surgical indication

### Comparison of SAPVE with those caused by other microorganisms

Compared to cases caused by other bacteria, patients with SAPVE were characterized by a slightly less advanced age (median age of 69 years versus 71 years, respectively; *p* = 0.019), higher incidence of early PVE, greater affinity for affecting the prosthetic valve in mitral position 52.5% vs. 35%; *p* < 0.001, and a higher percentage of chronic obstructive pulmonary disease (24.6% versus 18%; *p* = 0.022). Intracardiac complications and the development of pseudoaneurysm were more frequent in patients with PVE due to other microorganisms. In relation to the clinical course of the infection, a higher incidence of, persistent bacteremia, septic shock, stroke, and acute renal injury was observed. Hospital mortality was also higher (Table 1).

### Clinical characteristics of EVP due to methicillin-sensitive versus methicillin-resistant *S. aureus*

PVE due to MRSA accounted for 20.5% of cases. Sixteen patients with PVE due to MRSA (35.6%) had community-acquired PVE compared to 102 patients (58.6%) with PVE due to MSSA (*p* = 0.006). More patients suffered from active neoplasia and a high Age-Adjusted Charlson Comorbidity Index. There were no differences in clinical presentation, echocardiographic findings, clinical course, proportion of patients undergoing surgery, or mortality (Table 2). Patients with PVE due to MRSA presented persistent bacteremia more frequently than cases due to MSSA, although this difference did not reach statistical significance. Only in one of the cases due to MRSA did the patient receive cloxacillin monotherapy. It was given during the first day, before adjusting the treatment. In the remaining cases, patients received vancomycin or daptomycin from the time blood cultures were obtained.

**Table 2:** Characteristics of patients with PVE due to *Staphylococcus aureus* according to methicillin resistance

	MR *S. aureus* (*n* = 45)	MS *S. aureus* (*n* = 174)	*p*
Age. years (IQR)	69 (62–77)	68 (60–75)	0.358
Male gender	28 (62.2)	119 (68.4)	0.432
Community-acquired	16 (35.6)	102 (58.6)	0.006
Hospital-acquired	24 (53.3)	65 (37.4)	0.052
Non-nosocomial healthcare related	5 (11.1)	7 (4.0)	0.063
Early PVE (first year)	17 (37.8)	42 (24.1)	0.066
Site of infection			
Aortic	25 (55.6)	110 (63.2)	0.346
Transcatheter aortic valve implantation	1 (2.2)	6 (3.4)	0.677
Mitral	25 (55.6)	90 (51.7)	0.646
Tricuspid	1 (2.2)	1 (0.6)	0.300
Pulmonary	0	6 (3.4)	0.207
Cardiac implantable device	1 (2.2)	5 (2.9)	0.811
Comorbidity			
Chronic respiratory disease	14 (31.1)	40 (22.9)	0.260
Coronary disease	21 (46.7)	58 (33.3)	0.252
Cardiac insufficiency	23 (51.1)	80 (46.0)	0.539
Diabetes mellitus	17 (37.7)	49 (28.1)	0.210
Peripheral vascular disease	5 (11.1)	20 (11.5)	0.943
Cerebrovascular disease	9 (20.0)	20 (11.5)	0.118
Neoplasia	13 (28.9)	27 (15.5)	0.039
Chronic renal failure	13 (28.9)	57 (32.8)	0.620
Chronic liver disease	3 (6.7)	17 (9.7)	0.519
Congenital heart disease	3 (6.7)	11 (6.3)	0.933
Age-adjusted Charlson Comorbidity Index (IQR)	6 (4–7)	5 (3–7)	0.019
Echocardiographic findings			
Vegetation	31 (68.9)	127 (73.0)	0.584
Intracardiac complications	13 (28.9)	60 (34.5)	0.478
Valve perforation or rupture	2 (4.4)	3 (1.7)	0.273
Pseudoaneurysm	2 (4.4)	9 (5.2)	0.860
Perivalvular abscess	11 (24.4)	51 (29.3)	0.518
Intracardiac fistula	2 (4.4)	7 (4.0)	0.899
Clinical course			
Heart failure	24 (53.3)	76 (43.7)	0.246
Persistent bacteremia	10 (22.2)	26 (14.9)	0.240
Stroke	12 (26.6)	56 (32.1)	0.476
Embolism ^a^	6 (13.3)	37 (21.3)	0.233
Acute renal injury	26 (57.7)	105 (60.3)	0.754
Septic shock	17 (37.8)	60 (34.5)	0.680
Surgical indication	40 (88.9)	134 (77.0)	0.079
Surgery performed	22 (48.9)	66 (37.9)	0.281
Surgery indicated, not performed	18 (40.0)	68 (39.1)	0.910
Recurence	1 (2.2)	5 (2.8)	0.811
In-hospital mortality	19 (42.2)	86 (49.2)	0.389
First year mortality	21 (46.6)	95 (54.6)	0.342

MS: methicillin susceptible. MR: methicillin resistant. IQR: Interquartile range. ^a^ Excluding cases with stroke

### Characteristics of patients according to hospital mortality

The age of patients who died during admission (70 years; RIQ: 63–77 years) was higher than that of survivors (66 years, RIQ: 58–75 years; *p* = 0.037). These patients also presented chronic respiratory disease (48.5%), cardiac insufficiency (54.3%), chronic renal failure (41%) in a higher proportion than the survivors (20.1%, 40.4%, 23.7%, respectively; *p* < 0.05). Intracardiac complications appeared in a similar proportion in both groups (Table 3). The clinical course during admission was much worse in those who died with a higher proportion of patients presenting heart failure (59% versus 33.3%; *p* < 0.001), persistent bacteremia (21.9% versus 5.9%; *p* = 0.036), septic shock (56.2% versus 15.8%; *p* < 0.001, stroke (44.7% versus 19.3%; *p* < 0.001), systemic (non-CNS- embolism; 25.7% versus 14%; *p* = 0.030) and acute renal injury (71.4% versus 49.6%; *p* = 0.001). Finally, there were more cases with surgical indication (88.6% versus 71.1%; *p* = 0.001) and patients who did not undergo surgery even though it was indicated (47.6% versus 31.6%; *p* = 0.015; Table 3).

**Table 3:** Characteristics of patients with PVE due to *S. aureus* according to hospital mortality

	Non-survivors (*n* = 105)	Survivor (*n* = 114)	*p*	
Age. years (IQR)	70 (63–77)	66 (58–75)	0.037	
Male gender	70 (66.7)	77 (67.5)	0.890	
Community-acquired	55 (46.6)	63 (55.3)	0.669	
Hospital-acquired	44 (41.9)	45 (39.5)	0.719	
Non-nosocomial healthcare related	6 (5.7)	6 (5.3)	0.883	
Early PVE (first year)	27 (25.7)	32 (28.1)	0.695	
Site of infection				
Aortic	65 (61.9)	70 (61.4)	0.939	
Transcatheter aortic valve implantation	6 (5.2)	1 (0.9)	0.07	
Mitral	58 (55.2)	57 (50.0)	0.438	
Tricuspid	0	2 (1.8)	-	
Pulmonary	1 (1.0)	5 (4.4)	0.215	
Comorbidity				
Chronic respiratory disease	51 (48.5)	23 (20.1)	< 0.001	
Coronary disease	36 (34.3)	43 (37.7)	0.229	
Cardiac insufficiency	57 (54.3)	46 (40.4)	0.039	
Diabetes mellitus	35 (33.3)	31 (27.2)	0.322	
Peripheral vascular disease	14 (13.3)	11 (9.6)	0.392	
Cerebrovascular disease	13 (12.3)	16 (14.0)	0.718	
Neoplasia	17 (16.2)	23 (20.2)	0.446	
Chronic renal failure	43 (41.0)	27 (23.7)	0.006	
Chronic liver disease	8 (7.6)	12 (10.5)	0.456	
Congenital heart disease	6 (5.7)	8 (7.0)	0.694	
Age-adjusted Charlson Comorbidity Index (IQR)	5 (4–7)	4 (3–7)	0.054	
Microbiology				
MRSA	19 (18.1)	26 (22.8)	0.389	
Echocardiographic findings ^a^	96 (91.4)	93 (81.6)	0.034	
Vegetation	80 (76.2)	78 (68.4)	0.200	
Intracardiac complications	37 (35.2)	36 (31.6)	0.566	
Valve perforation or rupture	1 (1.0)	4 (3.5)	0.371	
Pseudoaneurysm	5 (4.8)	6 (5.3)	0.573	
Perivalvular abscess	31 (29.5)	31 (27.2)	0.702	
Intracardiac fistula	4 (3.8)	5 (4.4)	0.830	
Clinical course				
Heart failure	62 (59.0)	38 (33.3)	< 0.001	
Persistent bacteremia	23 (21.9)	13 (5.9)	0.036	
Stroke	46 (44.7)	22 (19.3)	< 0.001	
Time from stroke to surgery (days)	2 (0–23)	6 (2–18)	0.387	
Embolism ^b^	27 (25.7)	16 (14.0)	0.030	
Acute renal injury	75 (71.4)	56 (49.6)	0.001	
Septic shock	59 (56.2)	18 (15.8)	< 0.001	
Time from symptom onset to IE diagnosis (days)	3 (1–7)	5 (1–9)	0.532	
Rifampicin ^c^	62 (59.0)	78 (68.4)	0.149	
Gentamicin ^c^	34 (29.8)	22 (21.0)	0.133	
Surgical indication	93 (88.6)	81(71.1)	0.001	
Surgery performed	43 (41.0)	45 (39.5)	0.824	
Patients transferred for surgery	17 (14.9)	19 (18.1)	0.525	
Time from surgical indication to surgery (days)	5 (2–13)	2 (1–6)	0.006	
Surgery indicated, not performed	50 (47.6)	36 (31.6)	0.015	
Recurrence	0	6 (5.2)	-	

PVE: Prosthetic valve endocarditis. IQR: Interquartile range. MRSA: methicillin-resistant *S. aureus.*^a^ Echocardiographic findings: vegetation, abscess and/or moderate-severe regurgitation. ^b^ Excluding cases with stroke. ^c^ Patients that have received at least 1 dose of rifampin

The variables included in the multivariate analysis were age, onset or worsening of heart failure during admission, persistent bacteremia, acute renal failure, stroke, episodes of embolism other than located in central nervous system, and surgery not performed in cases in which it was indicated (Table 4). The variables independently associated with in-hospital mortality were age, calculated in years (OR:1.03, CI 95%: 1.00-1.05; *p* = 0.016), heart failure (OR:2.86, CI95% 1.53–5.32; *p* = 0.001), stroke (OR:3.53, CI 95%:1.79–6.96; *p* < 0.001), acute renal injury (OR:2.42 CI95%:1.28–4.58; *p* = 0.006), and surgery indicated. not performed (OR:2.01; CI 95%: 1.06–3.8; *p* = 0.030; Table 4).

**Table 4:** Multivariate analysis of prognostic factors in patients with PVE

	OR	CI 95%	*p*-value
Age, years	1.03	1.00–1.05	0.016
Heart failure	2.86	1.53–5.32	0.001
Persistent bacteremia	2.36	0.99–5.62	0.051
Acute renal injury	2.42	1.28–4.58	0.006
Surgery indicated. not performed	2.01	1.06–3.80	0.030
Stroke	3.53	1.79–6.96	< 0.001
Embolism	2.27	0.96–5.36	0.062

PVE: Prosthetic valve endocarditis. OR: Odds ratio. CI: Confidence interval

### Surgical treatment in patients with SAPVE

One hundred seventy-four patients (79.4%) presented surgical indication. Of these, only 88 patients (50.6%) underwent surgery. Patients who underwent surgery were characterized by lower age and comorbidity and a higher frequency of perivalvular abscess and other intracardiac complications (Table 1S in the supplementary material).

The reasons for indicating surgery in the patients who underwent surgery were heart failure due to severe valvular involvement (40 patients, 45.5%), persistent bacteremia (19 patients, 21.6%), perivalvular abscess (35 patients, 39.8%), intracardiac fistula (6 patients, 6.8%), pseudoaneurysm (4 patients, 4.5%) and high risk of embolism (8 patients, 9.1%). Some patients had more than one surgical indication. The percentage of patients with urgent and emergent indication was similar in both groups; 42.1% (19 patients) among those who survived versus 51.1% (51.1%) among those who died during hospital admission (p = 0.659), respectively. The reasons given in the remaining 86 patients for not performing surgery were extensive ischemic stroke (15 patients, 17.4%), cerebral hemorrhage (10 patients, 11.6%), marked hemodynamic instability (21 patients, 24.4%), advanced cirrhosis (4 patients, 4.7%), technical complexity (20 patients, 23.3%), rejection by the surgical team for futility (23 patients, 26.7%), unavailability of urgent surgery (1 patient, 1.2%) and refusal of surgery by the patient (7 patients, 8.1%), respectively. The main surgical indication when the procedure was not performed despite being indicated in patients who survived and those who died during admission were heart failure in 4 patients (11.1%) and 9 patients (18%; p = 0.352), risk of embolism 1 patient (2. 8%) and 3 patients (6%; p = 0.469), severe valvular regurgitation 3 patients (8.3%) and 1 patient (2%; p = 0.179), perivalvular complications 4 patients (11.5%) and 6 patients (12%; p = 0.834) and persistent bacteremia no patients and 6 patients (12%; p = 0.029), respectively”.

**Fig. 1:**
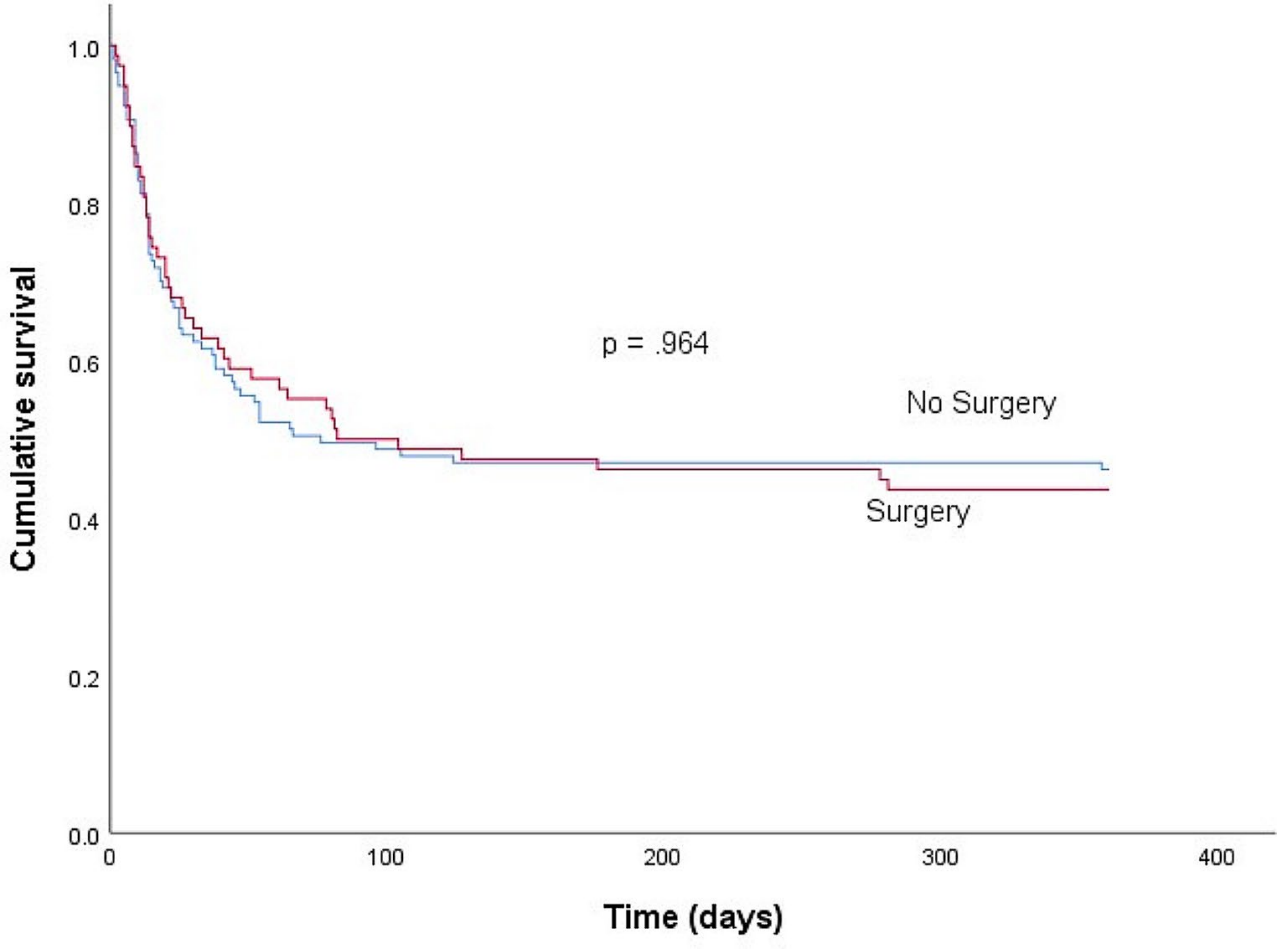
Survival of all patients with PVE due to S. aureus according to the performance of surgery, regardless of the existence of surgical indication

Finally, a comparison was performed between operated and non-operated patients using a propensity score (Table 5). This comparison showed that the performance of surgery per se in all patients with SAPVE, both those with and without surgical indications, was not associated with a reduction in in-hospital mortality (Fig. 1). The characteristics of the patients according to whether or not they underwent surgery are detailed in the supplementary material (Table 1S in the supplementary material). Table 2S (in the supplementary material) exhibits a multivariate analysis of variables related to in-hospital mortality in selected patients using the propensity score.

**Table 5:** Characteristics of patients with PVE due to *S. aureus* according to whether they underwent surgery using propensity score ^a^

Variables (%)	Non-surgery (79)	Surgery (79)	*p*
Age ≤ 66 years	34 (43.0)	34 (43.0)	1
Male gender	52 (65.8)	52 (65.8)	1
Early PVE (first year)	19 (24.1)	25 (31.6)	0.287
Site of infection			
Aortic	46 (58.2)	48 (60.8)	0.746
Mitral	47 (59.5)	46 (58.2)	0.872
Tricuspid	1 (1.3)	1 (1.3)	1
Pulmonary	1 (1.3)	1 (1.3)	1
Cardiac implantable device	3 (3.8)	1 (1.3)	0.62
Comorbidity			
Chronic respiratory disease	25 (31.6)	20 (25.3)	0.378
Coronary disease	25 (31.6)	29 (36.7)	0.311
Cardiac insufficiency	41 (51.9)	40 (50.6)	0.874
Diabetes mellitus	25 (31.6)	26 (32.9)	0.865
Peripheral vascular disease	12 (15.2)	7 (8.9)	0.221
Cerebrovascular disease	12 (15.2)	14 (17.7)	0.668
Neoplasia	20 (25.3)	12 (15.2)	0.113
Chronic renal failure	33 (41.8)	18 (22.8)	0.011
Chronic liver disease	12 (15.2)	3 (3.8)	0.014
Congenital heart disease	5 (6.3)	4 (5.1)	0.731
Age-adjusted Charlson Comorbidity Indez(IQR)	58 (73.4)	58 (73.4)	1
MRSA	18 (22.7)	22 (27.8)	0.464
Community-acquired	39 (49.4)	44 (55.7)	0.426
Hospital-acquired	34 (43.0)	31 (39.2)	0.628
Non-nosocomial healthcare related	6 (7.6)	4 (5.1)	0.513
New heart murmur	13 (17.1)	19 (26.0)	0.185
Echocardiographic findings ^b^	75 (94.9)	75 (94.9)	1
Vegetation	61 (77.2)	64 (81.0)	0.557
Intracardiac complications	23 (29.1)	35 (44.3)	0.048
Valve perforation or rupture	1 (1.3)	4 (5.1)	0.367
Pseudoaneurysm	4 (5.1)	3 (3.8)	0.565
Perivalvular abscess	20 (25.3)	33 (41.8)	0.028
Intracardiac fistula	2 (2.5)	6 (7.6)	0.276
Clinical course			
Heart failure	38 (48.1)	38 (48.1)	1
Persistent bacteremia	13 (16.5)	17 (21.5)	0.417
Stroke	23 (29.5)	30 (38.0)	0.261
Embolism ^c^	15 (19.0)	14 (17.7)	0.837
Acute renal injury	44 (56.4)	51 (64.6)	0.296
Septic shock	35 (44.3)	23 (29.1)	0.048
In-hospital mortality	41 (51.9)	39 (49.4)	0.750
First year mortality	45 (56.9)	42 (53.1)	0.631
Rifampicin ^d^	50 (63.3)	47 (59.5)	0.624

MRSA: methicillin-resistant *S. aureus.*^a^ Adjusted for gender, age ≤ 66 years, age-adjusted Charlson Index ≥ 4 points and for the presence of echocardiographic findings. ^b^ Echocardiographic findings: vegetation, abscess and/or moderate-severe regurgitation. ^c^ Excluding cases with stroke. ^d^ Patients that have received at least 1 dose of rifampin

## Discussion

Patients with SAPVE in this study were characterized by advanced age and marked comorbidity. A considerable percentage of this infection was acquired in the context of health care. The clinical course was usually complicated, and mortality was high. It should also be noted that a significant proportion of patients did not undergo surgery despite having a surgical indication.

### Comparison of SAPVE with those caused by other microorganisms

The series presented in this article is the largest published to date, with SAPVE cases accounted for 16.7% of all cases of PVE. This percentage was lower than that found in series of PVE [9, 11], but similar to those of other studies [3, 7]. Another characteristic of our patients was the high proportion of cases diagnosed during the first year after valve implantation, as previously reported [21].

It should be noted that chronic respiratory diseases were more frequent in patients SAPVE. Obstructive pulmonary disease has been considered a risk factor for bacteremia from this species [22], probably because of the frequent need for hospital treatment and use of intravenous lines. Also noteworthy was the greater tendency of S. *aureus* to affect prostheses in mitral position, compared to PVE due to other microorganisms. Although this result has not been obtained in some studies [7, 10], other studies have found certain affinity of *S. aureus* to affect prostheses in the mitral position [3], but no explanation for this possible association has been found.

The most important difference between the two groups was the higher mortality associated with cases due to *S. aureus* [8, 21, 23]. It should be noted that 30% of the patients developed a stroke, a figure similar to that of previous studies [3, 24]. The higher frequency of persistent bacteremia, septic shock and stroke are factors clearly related to patient prognosis, as has been observed in other studies [2, 7–11, 25]. Despite the higher clinical severity, fewer patients with SAPVE were treated surgically compared to patients with PVE caused by other microorganisms [3, 6, 8, 9, 26].

### Clinical characteristics of PVE due to methicillin-sensitive versus methicillin-resistant *S. aureus*

Studies that have compared the characteristics of PVE caused by MRSA in relation to those caused by MSSA have been few and with a relatively limited number of patients [3, 6–8]. The proportion of PVE caused by MRSA was 21% in our study, which is within the range of other published series (6–41%) [3, 6, 9, 11, 26, 27]. Differences in the characteristics of the patients studied and the time in which the studies were performed may account for the observed variability. These patients were also characterized by a more pronounced comorbidity, as well as by the frequent presence of an active neoplasm, circumstances that could be associated with a higher risk of colonization or MRSA infection [6, 28]. As expected, most of the cases due to MRSA were acquired in relation to health care. We also observed a certain tendency (without statistical significance) for PVE due to MRSA to appear during the first months after valve prosthesis implantation, as has been detected in other series [3].

In a series of patients with NVE and PVE due to *S. aureus*, a higher proportion of persistent bacteremia was observed in 26% of cases and was significantly more frequent in patients with MRSA [8]. Other variables related to this infectious complication were the nosocomial origin of IE, surgical intervention in the previous 6 months, the presence of a catheter and surgical site infection. In our series there were more cases of persistent bacteremia when the infection was caused by MRSA (22% versus 15%), but the difference did not reach statistical significance. There was also no evidence of differences in the risk of developing stroke, as was observed in a series of IE (native and prosthetic) caused by *S. aureus* [27].

In some series that included both PVE and NVE, there has been evidence of higher mortality in patients infected with MRSA, which has been related to less adequate treatment, in some cases because surgery was refused [8, 27]. In another study, however, no relevant differences were found [6]. Although a higher mortality in patients with MRSA bacteremia than MSSA has been demonstrated in several studies [4, 5], we believe that the similar performance of surgery in both groups could justify the similar in-hospital mortality in our patients.

### Characteristics of patients according to hospital mortality

The high in-hospital mortality, which has been repeated in different studies on SAPVE, should be noted [3, 7, 9, 11]. This dramatic result should encourage us to investigate SAPVE in greater depth to develop strategies to reduce it. As expected, we found that age was associated with in-hospital mortality, a result similar to that found in other studies [9, 10]. It should be noted that in one of these studies all patients under 50 years of age who did not undergo surgery survived hospital admission [10].

In several previous studies stroke was identified as the main prognostic factor [9, 24]. In a recent investigation, 64% of patients with stroke died (especially when there was a significant hemorrhagic component) [3]. Ischemic stroke and cerebral hemorrhage increase morbidity, largely because they may hinder (or prevent) early valve replacement in these patients [29].

Heart failure is a very frequent complication in patients with SAPVE [3, 7, 10, 29], with an incidence higher than that found in patients with PVE due to other microorganisms [11]. This complication is usually due to extensive valve damage and is the most frequent cause of both surgical indication and the patient’s own death [3, 9, 26]. In our series, the percentage of deceased patients with heart failure (62%) was twice that of survivors (33%), indicating its strong association with the patient’s prognosis. Interestingly, a study by the ICE (International Collaboration on Endocarditis) showed that patients whose indication for surgery was a valvular or paravalvular complication underwent surgery more frequently than when the indication is heart failure (1).

Renal failure is another complication that appears very frequently when the bacteria product of PVE is *S. aureus* [3, 11]. Renal hypoperfusion, toxicity of certain drugs, renal embolism and immunological complications are frequent complications in cases of SAPVE and are associated with renal failure [3, 24, 30]. This complication has been associated with mortality in studies on PVE of diverse etiology [21] and showed a trend close to statistical significance in previous studies on SAPVE [7, 24].

### Surgical treatment in patients with SAPVE

One of the characteristics repeatedly observed in the different published series is the small number of patients who undergo surgery [1, 3, 7, 9, 11, 23, 26, 29, 31]. The decision to forego surgery in patients with surgical indication has a significant impact on prognosis [9]. Although the mortality of patients with surgical indication who underwent surgery (48.9%) was lower than those who did not undergo surgery, (58.1%, *p* = 0.220) this difference did not reach statistical significance in our series. However, other studies have found a greater difference in the prognosis of both groups (28.6% versus 53.3%) [29]. It should be also noted that the time elapsed between the indication for surgery in our patients and its performance was greater in patients who died. This suggests that the performance of the intervention should not be delayed if the best results are to be obtained in terms of patient prognosis [9]. Obviously, the type of indication (emergent, urgent, elective) should influence the speed with which surgery is performed. The high mortality, even in surgically operated patients, may have been related to a poor baseline clinical situation, determined by a rather advanced age and a high degree of comorbidity, which could lead to a higher mortality in the context of any eventual complication. Although patients older than 65 years tend to have a worse prognosis due to comorbidities, we consider that age alone should not be such a significant factor to exclude surgery [32, 33]. The reason most frequently given for excluding surgery was the existence of an ischemic stroke or intracerebral hemorrhage. A proper assessment of the type of stroke (ischemic versus hemorrhagic) and its extent is essential before discouraging surgery [3, 34, 35]. Severe systemic infection or greater surgical complexity in these patients could also be related to refusal of surgery [3, 9, 10]. When analyzing the main surgical indication in patients who did not undergo surgery, it is observed that all patients whose indication was the persistence of bacteremia died, which can be related to the virulence of *S. aureus* and the consequences of not eliminating the intravascular infectious focus [9, 10].

Strategies to reduce the number of patients denied surgery may include better patient education about treatment options, adherence to recommended surgical timelines (emergent, urgent or elective) and facilitating transfers to hospitals with experience in complex surgery [8, 9, 11, 23]. Although the optimal moment to perform surgery is an unresolved issue [9], the observation by Sáez et al. that renal failure, stroke of emboligenic origin and septic shock are frequent during the first days after the diagnosis of renal failure, reinforce the need for surgery to be performed as soon as possible [3].

One of the most debated issues in recent years is whether surgery should be recommended for all patients. Current European and American endocarditis guidelines agree that the virulence of *S. aureus* determines the surgical indication in these patients [1, 15]. In fact, a recent meta-analysis analyzing five studies on the prognosis of patients with SAPVE showed a lower mortality with surgery [36]. John et al., also observed lower mortality in cases of PVE due to SA that were operated on during antibiotic treatment [26]. In this article, however, no distinction was made as to whether or not surgery was indicated according to clinical guidelines. Other studies argue that in order to recommend surgical treatment, it is necessary to consider the characteristics of the patients, since there is a group of patients without relevant cardiac or systemic complications whose evolution can be favorable without surgery [9, 10]. In this regard, Lalani et al. did not find that surgery per se improved prognosis in a series on PVE of various etiologies [23]. Our study supports that surgery should be recommended only in cases with a clear indication due to hemodynamic status, lack of infection control or high risk of embolism [1, 15]. A randomized trial of early surgery versus indication-based surgery would be most appropriate, but we consider that it would be a difficult study to carry out.

### Limitations

Our study has several limitations, such as the fact that it was a multicenter study with possible differences in the type of patient and in the selection of treatment. It should also be noted that many patients were referred from hospitals without cardiac surgery, which could have influenced the etiology and certain characteristics of the patients studied. More severe or milder cases might have been transferred less frequently because surgical intervention can be ruled out at the outset. However, these differences should not be very important considering the fluid communication and adequate coordination between hospitals without cardiac surgery and referral hospitals.

## Conclusions

We consider that our study provides relevant information on SAPVE, such as the marked clinical severity and mortality, the limited differences between cases caused by MRSA or MSSA, and the relationship between in-hospital mortality and not performing surgery in patients with surgical indications according to the clinical guidelines. This results which should serve as a stimulus for better identification of patients who could benefit from surgery. On the other hand, this study confirms that cardiac surgery in all patients with SAPVE, regardless of whether or not there is a surgical indication, is not associated with a reduction in in-hospital mortality.

## Electronic supplementary material

Below is the link to the electronic supplementary material.

Supplementary Material 1

## Data Availability

No datasets were generated or analysed during the current study.

## Abbreviations

IE: Infective endocarditis
IQR: Interquartile range
MRSA: Methicillin-resistant S. aureus
MSSA: Methicillin-sensitive *S. aureus*
PVE: Prosthetic valve endocarditis
SAPVE: Prosthetic valve endocarditis due to *S. aureus*
